# SARS‐CoV‐2 in patient with protein C deficiency: A case report

**DOI:** 10.1002/ccr3.8030

**Published:** 2023-10-15

**Authors:** Akram Sadat Ahmadi, Nazanin Zahra Shafiei‐Jandaghi, Kaveh Sadeghi, Vahid Salimi, Ahmad Nejati, Talat Mokhtari Azad, Jila Yavarian

**Affiliations:** ^1^ Department of Virology, School of Public Health Tehran University of Medical Sciences Tehran Iran; ^2^ Research Center for Antibiotic Stewardship and Antimicrobial Resistance Tehran University of Medical Sciences Tehran Iran

**Keywords:** coagulation, COVID‐19, protein C deficiency, SARS‐CoV‐2

## Abstract

In SARS‐CoV‐2 pandemic different disorders in coagulation pathways in COVID‐19 patients were reported. We described a 44‐year‐old female with COVID‐19 and protein C deficiency history. She did not show any coagulation disorder during her disease course. Complete genome sequencing of SARS‐CoV‐2 was performed and some mutations identified and compared with Wuhan strain. Besides hospitalized patients, in COVID‐19 outpatients with low concentration of protein C, early prescription of an anticoagulant such as heparin could be helpful in prevention of venous thromboembolism or pulmonary embolism.

## Graphical abstract

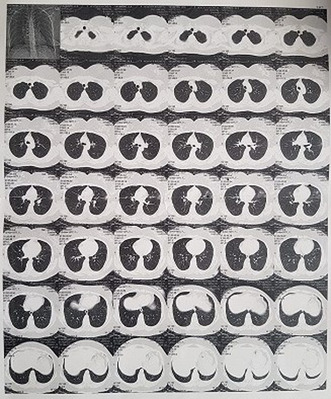

## 1 INTRODUCTION

The novel coronavirus, severe acute respiratory syndrome coronavirus‐2 (SARS‐CoV‐2), in the current pandemic is created many complications for patients all over the world. After reporting the first cases of COVID‐19 in February 2020 in Iran we faced different forms of illness and complicated status in our country.^1^ SARS‐CoV‐2 can cause different manifestations like coagulation disorders and thrombotic complications in patients. The pathogenesis of the COVID‐19‐induced coagulopathy has not yet been fully elucidated. Thrombophilia is defined as the increased propensity of blood for development of the thrombose, that is proposed as a cause of hypercoagulability. It is possible that people with thrombophilia are susceptible to death due to COVID‐19. Therefore, for prophylactic purpose and reduction of the risk of death low‐weight heparin is suggested for administration.^2^ Protein C has anticoagulant role in coagulation pathway. The deficiency of protein C disturbs the balance between procoagulant and anticoagulant proteins, therefore, it may cause venous thromboembolism (VTE) and pulmonary embolism (PE).^3^, ^4^ As coagulopathy is one of the COVID‐19 complications, people with protein C deficiency may be susceptible to thrombotic complications.^5^ Some evidence reported severe outcome related to SARS‐CoV‐2 infection and hypercoagulable disorder in COVID‐19 patients.^6^, ^7^, ^8^, ^9^

Here we reported a COVID‐19 case with protein C deficiency which with appropriate prophylaxis, she had not experienced thrombotic complications.

## 2 CASE HISTORY

A 44‐year‐old female who had positive SARS‐CoV‐2 PCR test (Threshold Cycle Ct:30) on 4 November 2020 with symptoms of fatigue and lethargy. She had been in direct contact with a COVID‐19 patient in 28 October 2020. Her SARS‐CoV‐2 PCR test was negative on 1 November 2020. After SARS‐CoV‐2 infection confirmation on 4 November, she was isolated with complete bed rest. The SARS‐CoV‐2 genomic sequencing data showed the circulation of GH clade from October to November 2020 in Iran.^10^

## 3 DIFFERENTIAL DIAGNOSIS, INVESTIGATIONS, AND TREATMENT

**FIGURE 1:**
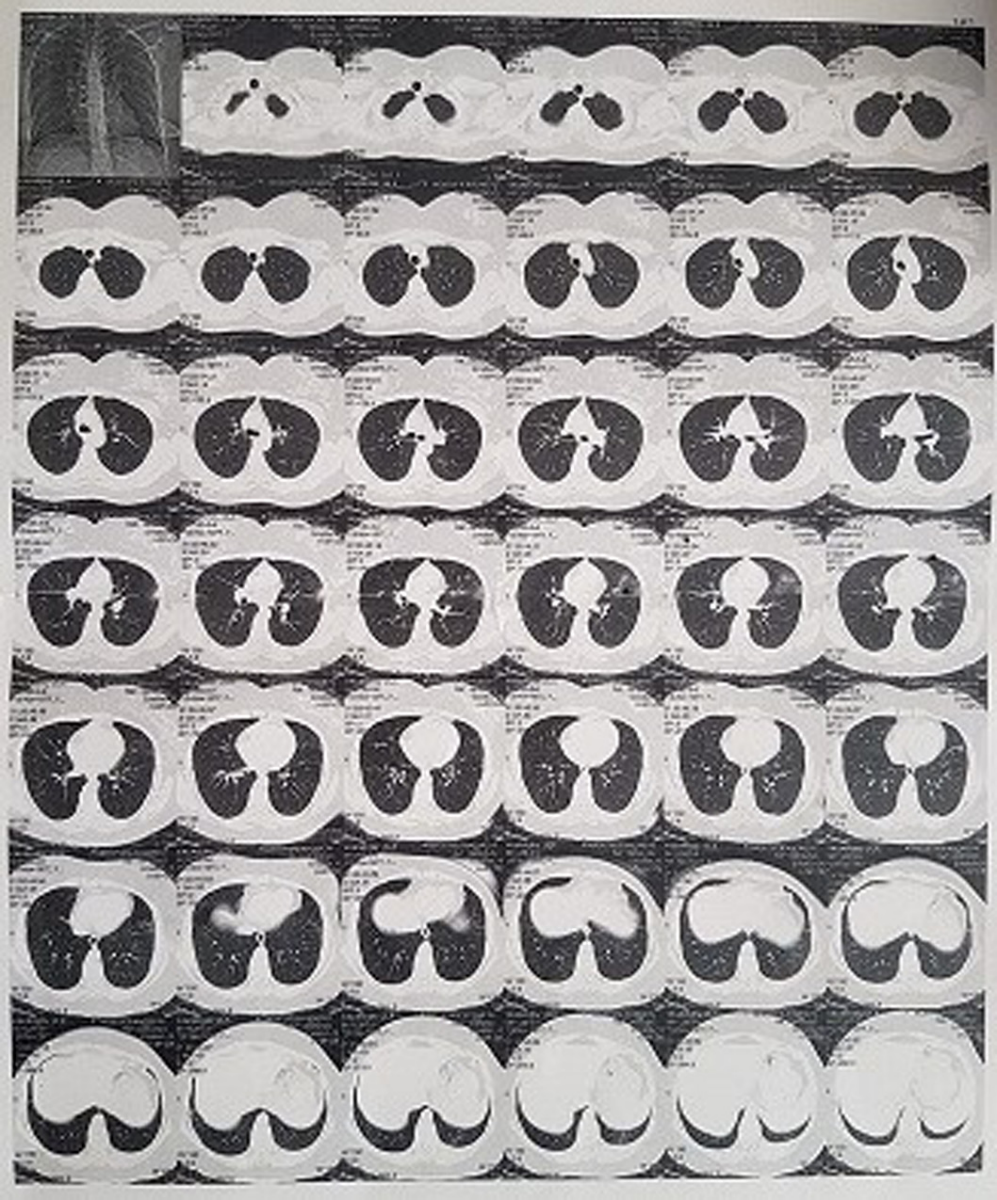
Computed tomography (CT) scan in COVID‐19 patient with unilateral lung involvement.

The patient's primary laboratory tests were normal. On the third day of illness fever was 38°C and the patient's main complaint were fatigue, lethargy, insomnia, restlessness, and increased appetite with SPO_2_; 92%–95%. Based on the history of protein C deficiency in patient, she received heparin 6000 unit every night since the first day of diagnosis. As a viral therapy since 5th November, Sovodak tablet (Sofosbuvir plus Daclatasvir) was ordered daily until 10 days. Vitamin C (500 mg), vitamin D (1000 mg), zinc plus and melatonin tablets daily and famotidine 40 mg BD were consumed. On the Day 9, patient suffered from shortness of breath. Computed tomography (CT) scan was performed which she had a unilateral lung involvement (Figure 1) and she was hospitalized on 14th November. On the day of hospitalization (Day 11), her PCR test was positive with Ct:25. In hospital she received five doses of remdesivir for 5 days. Dexamethasone injection and pantoprazole tablets BID were ordered. Electrocardiography (ECG) and echocardiography were performed due to acute chest pain which were normal. On Day 15, at the time of discharge from the hospital, PCR test was negative. During the acute phase of the diseases, liver enzymes were raised but after 1 month became normal. The patient's menstrual period was delayed for 18 days due to COVID‐19. Laboratory findings include coagulation factors, biochemistry and complete blood count (CBC) were shown with comparison the results in different days of the illness in Table 1. It is worth mentioning that the D‐Dimer level was reported negative on 5 days after infection.

**TABLE 1:** Laboratory findings of the COVID‐19 patient with protein C deficiency.

Marker	Level		Unit	Normal value
	2nd day	11th day		
WBC	4.8	12.1	×1000/mm^3^	4.1–10.1
RBC	4.63	4.41	Million/nm m	4.2–5.8
Hemoglobin	13.6	14.0	g/dL	12–16
MCV	88.9	89.6	fL	77–94
MCH	30.0	30.2	pg	26–33
MCHC	34.7	33.7	g/dL	31–37
RDW‐CV	13.3	13.0	fL	‐
Platelets	277	212	×1000/mm3	150–400
Neutrophil	84.3	55	%	‐
LYMPH	9.0	38	%	‐
ESR	2	14	mm/h	Female 86–100 years < 42
CRP	2	20	mg/L	Adult <6.0
AST	23	98	U/L	Adult Female <31
ALT	22	201	U/L	Adult Female <31
ALP	152	204	U/L	Adult 70–306
LDH	207	612	U/L	<480
PT	11.5	‐	s	‐
PT control	11.5	‐	s	‐
PT activity	100	‐	%	‐
INR	1	‐	‐	1–1.4
APTT	34	‐	Sec	30–42
Protein C	58	‐	%	>5 years 70–150
Protein S	96	‐	%	>1 years 65–125
Anti‐Thrombin III	100	‐	%	Adult 80–125
NT‐PRO‐BNP	163	‐	μg/L	18–44 years: 96
APCR (Leiden factor)	2.87	‐	Ratio	= or >2.0
25‐OHVitamin D	68.4	‐	ng/mL	Desirable for general health >30

For SARS‐CoV‐2 mutation analysis, full genome sequencing was performed on the patient's first sample with Illumina NextSeq at National Influenza Center, Tehran University of Medical Sciences. In SARS‐CoV‐2 genome sequencing, GH clade (Spike D614G and NS3 Q57H) was observed and some mutations in comparison with hCoV‐19/Wuhan/WIV04/2019 strain were identified.^11^ (Table 2).

**TABLE 2:** Results of SARS‐CoV‐2 full genome sequencing in COVID‐19 patient with protein C deficiency.

Virus Name	hCoV‐19/Iran/Tehran‐4/2020
Accession ID	EPI_ISL_862079
Type	Beta coronavirus
Clade	GH
Pango Lineage	B.1.36 (Version 2021‐5‐12)
AA Substitutions	Spike‐D614G, Spike‐1210del, Spike‐ Q314R, N‐ P13T, N‐S194L, NS3‐Q57H, NSP1‐S135N, NSP2‐A510V, NSP3‐G733R, NSP12‐ L638F, NSP12‐P323L

## 4 DISCUSSION

In this report, we introduced a COVID‐19 patient with protein C deficiency. During the SARS‐CoV‐2 pandemic, coagulation abnormalities in COVID‐19 patients were reported, and proposed that COVID‐19 patients with protein C deficiency faced with a risk factor for severe disease,^6^, ^12^ but there is no describing evidence about different symptoms and other complications in COVID‐19 patients with protein C deficiency. Maqbool et al. reported a case with heterozygous protein C deficiency presented PE and myocardial infraction.^4^ A recent research reported that protein C and S deficiencies are current undiagnosed thrombophilia in the COVID‐19 patients with severe outcome when compared with non‐severe ones. It is possible that these undiagnosed disorders in the high‐risk COVID‐19 patients have a role in severe outcome and development of thrombosis.^13^ Elshafie et al. reported reduction in protein C and S activities in COVID‐19 patients in comparison with healthy populations.^14^ An investigation documented the relationship between low levels of protein C at the time of admission with severity and mortality of hospitalized COVID‐19 individuals.^12^

Several research stated that treatment with heparin (dalteparin, enoxaparin, etc.) is cardinal therapy in COVID‐19‐induced coagulopathy such as VTE and PE.^15^, ^16^ Tang et al. recommended treatment with heparin following coagulopathy in severe COVID‐19 patients and stated that it is related to better prognosis.^17^ They suggested beneficial properties for anticoagulants treatment primarily with low molecular weight heparin (LMWH) in COVID‐19 patients which have the elevated D‐Dimer and sepsis‐induced coagulopathy (SIC). Noteworthy, the other studies have proposed non‐coagulant effects for heparin especially anti‐inflammatory roles in COVID‐19 patients.^18^

The asymptomatic and undiagnosed thrombophilia in some situations such as hyper‐estrogenic conditions (pregnancy and postpartum) might result in development of different disorders.^13^ A study had been stated that protein C deficiency plays role in fetal losses in pregnancies.^19^ In agreement with the mentioned phenomenon, our case due to protein C deficiency, had a history of intrauterine fetal death (IUFD) on her first pregnancy when protein C deficiency was undiagnosed and during the second pregnancy heparin was used with a healthy outcome.

In this report, the COVID‐19 patient with protein C deficiency had lung involvement and liver enzymes abnormalities. Excluding the protein C deficiency, other coagulation parameters were normal, but for prophylaxis of possible complications, she started taking heparin since the first day of her disease.

In conclusion, following our observations in COVID‐19 patients with protein C deficiency, besides hospitalized patients, it is better to start an anticoagulant also in outpatients under physician supervision and recommendation for prophylaxis of possible VTE or PE.

## AUTHOR CONTRIBUTIONS

**Akram Sadat Ahmadi:** Writing – original draft. **Nazanin Zahra Shafiei‐Jandaghi:** Investigation; methodology. **Kaveh Sadeghi:** Data curation; methodology. **Vahid Salimi:** Methodology. **Ahmad Nejati:** Methodology. **Talat Mokhtari Azad:** Supervision. **Jila Yavarian:** Conceptualization; project administration; writing – review and editing.

## FUNDING INFORMATION

This study was partly supported by School of Public Health, Tehran University of Medical Sciences under Grant No. 1400‐1‐99‐53051.

## CONFLICT OF INTEREST STATEMENT

All of the authors have no conflict of interest to declare.

## CONSENT

Written informed consent was obtained from the patient to publish this report in accordance with the journal's patient consent policy.

## Data Availability

The data used in this study are available from the corresponding author upon reasonable request.
